# Right hippocampal volume and attenuated psychotic symptoms distinguish subgroups of youth with common patterns of temporoparietal effective connectivity

**DOI:** 10.1007/s00429-026-03161-1

**Published:** 2026-08-08

**Authors:** Katrina Aberizk, Benson S. Ku, Hengyi Cao, Charles H. Schleifer, Jean M. Addington, Carrie E. Bearden, Kristin S. Cadenhead, Tyrone D. Cannon, Ricardo E. Carrión, Matcheri Keshavan, Daniel H. Mathalon, Diana O. Perkins, William S. Stone, Scott W. Woods, Elaine F. Walker

**Affiliations:** 1 Department of Psychiatry and Behavioral Health, Clinical and Translational Science Institute, The Ohio State University College of Medicine, 1960 Kenny Rd, Columbus, OH 43210 USA; 2 Department of Psychology, Emory University, Atlanta, GA USA; 3 Department of Psychiatry and Behavioral Sciences, Emory University School of Medicine, Atlanta, GA USA; 4 Institute of Behavioral Science, Feinstein Institutes for Medical Research, Northwell Health, Glen Oaks, NY USA; 5 Departments of Psychology and Psychiatry and Biobehavioral Sciences, University of California, Los Angeles, CA USA; 6 Department of Psychiatry, University of Calgary, Calgary, AB Canada; 7 Department of Psychiatry, University of California, San Diego, CA USA; 8 Departments of Psychology and Psychiatry, Yale University, New Haven, CT USA; 9 Department of Psychiatry, Donald and Barbara Zucker School of Medicine, Northwell Health, Glen Oaks, NY USA; 10 Beth Israel Deaconess Medical Center, Harvard Medical School, MA Boston, USA; 11 Department of Psychiatry, Harvard Medical School, Cambridge, MA USA; 12 Department of Psychiatry, University of California, San Francisco, CA USA; 13 Department of Psychiatry, University of North Carolina at Chapel Hill, Chapel Hill, NC USA

**Keywords:** GIMME, Hippocampus, NAPLS, Psychosis, Structure–function

## Abstract

Associations between patterns of brain connectivity and brain structural features have transdiagnostic relevance to psychopathology. There is considerable evidence for disruptions to the hippocampus and temporoparietal brain systems in psychotic disorders. The present study examines structure–function relationships—specifically, the relation of hippocampal volume with patterns of temporoparietal effective connectivity—in youth at clinical high-risk for psychosis (CHR-P) and healthy controls (HCs). Participants at CHR-P and HCs completed clinical symptom measures and magnetic resonance imaging at baseline (*n* = 388, 42.5% female, age = 19.8 ± 4.2) in the second cohort of the North American Prodrome Longitudinal Study. Group Iterative Multiple Model Estimation established a common functional network of temporoparietal effective connectivity in the full sample. Next, supervised (assuming the CHR-P and HC groups represent classes) and unsupervised (data-driven) clustering procedures interrogated effective connectivity parameters relevant to subsamples. Mean difference tests by unsupervised cluster membership determined whether clusters formed based on temporoparietal effective connectivity differed in hippocampal volume. Clusters were also compared on representation of CHR-P and positive and negative symptom totals to determine whether unsupervised clustering recovered clinical features. Unsupervised clustering generated two clusters that differed significantly in right hippocampal volume and attenuated positive and negative symptoms with small-to-medium effect sizes. The cluster demonstrating reduced right hippocampal volume reported greater symptoms. Unsupervised clustering did not recover diagnostic groups. Findings are consistent with literature indicating the transdiagnostic relevance of hippocampal volume to organizational properties of brain functional networks and patterns of temporoparietal connectivity.

**Supplementary Information:**

The online version contains supplementary material available at https://doi.org/10.1007/s00429-026-03161-1.

## Introduction

Separate lines of evidence have recently converged in showing significant statistical relationships between patterns of brain connectivity and brain structural features (Fotiadis et al. 2024; Hong et al. 2023; Sarwar et al. 2021). Some investigations in this area of research have focused on people with psychosis or at clinical high-risk for psychosis (CHR-P) (Aberizk et al. 2023; Prasad et al. 2023; Harms et al. 2013). Studies of CHR-P involve the administration of standardized, structured interviews that rate the severity of attenuated psychotic symptoms (e.g., odd beliefs and perceptions) that typically precede frank psychosis (Walker et al. 2013). In the literature on structure–function coupling in psychosis, the hippocampus has received considerable attention because both hippocampal structural and functional alterations are observed across the spectrum of psychotic psychopathology.

Reduced hippocampal volume (HV) is among the most well-replicated brain morphological features of psychotic disorders (Gutman et al. 2022; van Erp et al. 2016); it is also observed in first episode psychosis (FEP) and most pronounced in those whose symptoms do not remit within two years of illness onset (McHugo et al. 2020). Considered together, evidence suggests that reductions in HV are well-established in psychotic disorders (Gutman et al. 2022), including in FEP (McHugo et al. 2020), but their timing relative to the onset of psychosis remains unresolved. Meta-analyses do not provide evidence for significant baseline differences in HV between people at CHR-P and HCs (Walter et al. 2016) or between people at CHR-P who develop psychosis within two years and those who do not (Hinney et al 2021). More broadly, reduced HV is not prognostic of psychotic illness. It is a common feature in people with bipolar and major depressive disorders (Brosch et al. 2022), neuroendocrine disease (Burkhardt et al. 2015), and thought disorder (Stein et al. 2021). Longer-term follow-up (> 5 years) of people at CHR-P shows that approximately 20% of youth at CHR-P will develop a psychotic disorder, 60% will not remit from CHR-P status, and 70% of those who do not develop psychosis will develop an affective disorder (Cadenhead et al. 2025). Thus, CHR-P status encompasses a spectrum that warrants further investigation.

With respect to function, hippocampal hyperactivity is a risk factor for psychotic disorder and an indicator of acute psychotic illness (Heckers and Konradi 2015; McHugo et al. 2022; Modinos et al. 2021; Schobel et al. 2013). It remains unknown whether hippocampal hyperactivity in acute and prodromal stages of psychosis is associated with altered hippocampal *connectivity*. We are aware of one previous study that demonstrated hippocampal hyperactivity in FEP relative to HCs, but normal hippocampal connectivity with brain-wide functional networks (e.g., default mode), providing the first direct evidence that hippocampal hyperactivity may not translate to aberrant functional network dynamics (McHugo et al. 2015). However, the authors of that previous study demonstrated that their results were sensitive to the granularity and location of independent components of brain network connectivity and suggested that future investigations consider individual brain areas as seed regions of interest (ROIs). Indeed, as the literature on hippocampal structure–function coupling has burgeoned, many investigations have adopted a seed-based ROI approach in functional connectivity (FC) analysis. FC is a measure of covariance in brain regional blood oxygenation level-dependent (BOLD) signal, measured during functional magnetic resonance imaging (fMRI), that has informed decades of research on normative brain function and aberrations in psychiatric disorders (Fornito et al. 2012; Samudra et al. 2015).

In healthy adults, seed ROI-based FC between the temporoparietal junction and global default mode network (DMN) has been positively associated with HV; specifically, HV was a stronger predictor of DMN-temporoparietal FC than age (De Marco et al. 2019). In adults with schizophrenia (SZ), the most severe psychotic disorder, HV is positively associated with brain activation in prefrontal regions implicated in executive functions during working memory tasks (Harms et al. 2013). In first-degree relatives of people with SZ, reduced HV has been associated with reduced suppression of DMN activity during working memory and vigilance tasks, which suggests that reduced HV may be associated with extraneous neural activity during executive functioning demands (Seidman et al. 2014). Considered together, the emergent pattern of findings regarding hippocampal structure–function coupling implicates temporoparietal areas of the DMN, an apex network responsible for coordinating multimodal integration and cognitive processing less bound to real-time sensory input (Buckner and DiNicola 2019; Fornito et al. 2011). These temporoparietal ROIs are implicated in self-referential thinking processes including episodic memory encoding and retrieval, feelings of agency, judgments about the present, and evaluations of the self (Andrews-Hanna et al. 2010; Smith et al. 2018).

We recently published findings from the first study of hippocampal structure–function coupling during resting-state fMRI (rsfMRI) including people at CHR-P and HCs. First, we demonstrated significant inverse relations of HV and hippocampal FC with the inferior parietal lobule and thalamus during rest in both participant groups. Next, we showed that CHR-P status moderated relations between HV and hippocampal FC with temporoparietal ROIs (e.g., inferior parietal lobule, superior temporal pole), thalamus, and posterior cingulate cortex (Aberizk et al. 2023). The present study aims to advance the findings of Aberizk et al. (2023) by leveraging an effective connectivity procedure that preserves the complete BOLD signal time series to estimate both directional and time-lagged relationships among BOLD fluctuations in a functional network, which static FC procedures do not measure (Gates and Molenaar 2012).

Specifically, we employ supervised and unsupervised clustering procedures available within the selected effective connectivity approach (Henry et al. 2019; Gates et al. 2017) to explore, respectively: *1)* whether CHR-P and HC “classes” shared common patterns of temporoparietal effective connectivity over and above connectivity parameters estimated in the full sample, and *2)* self-organization of the sample (without constraint by subgroup number or identity) driven by commonalities in individual patterns of temporoparietal effective connectivity. Two competing hypotheses were plausible concerning the outcome of unsupervised clustering. Relative to HCs, people at CHR-P have shown large-scale functional brain network aberrations (Cao et al. 2020), as well as thalamocortical (Cao et al. 2018) and temporal lobe hyperactivity (Davies et al. 2024; Dutt et al. 2015) relative to HCs, so the unsupervised clustering may recover the diagnostic groups.

Alternatively, consistent with extant evidence demonstrating that temporoparietal *FC* is associated with HV in healthy people (De Marco et al. 2019), people at CHR-P (Aberizk et al. 2023), and first-degree relatives of adults with SZ (Seidman et al. 2014), temporoparietal *effective* connectivity may be more similar among people with similar HV. FC estimates are known to be biased by their lack of consideration for time-lagged information (Arbabshirani et al. 2014), so if temporoparietal *effective* connectivity is shown be associated with HV, then this would lend support to the literature suggesting that alterations in HV have implications for patterns of brain connectivity. To examine support for these competing hypotheses in the present study, unsupervised clusters formed on the basis of common patterns of temporoparietal effective connectivity were compared on proportion of individuals at CHR-P as well as positive and negative symptom totals; further, in addition to HV, clusters were compared on bilateral amygdala, nucleus accumbens, and thalamus volumes post hoc because reductions in those regional volumes are the most well-replicated brain morphologic findings in adults with psychosis after reductions in HV (Gutman et al. 2022; van Erp et al. 2016).

## Methods and materials

### Sample

The present study included participants from the second phase of the North American Prodrome Longitudinal Study (NAPLS 2) who completed structural imaging and an rsfMRI scan at baseline (*n* = 388) including 142 HCs and 246 people at CHR-P with data that passed quality assurance. All at CHR-P met criteria for attenuated positive symptom syndrome on the Structured Interview for Psychosis-risk Syndromes (SIPS; McGlashan et al. 2010) at baseline. The aims, recruitment methods, and inclusion criteria for NAPLS 2 are detailed in Addington and colleagues (2012). Briefly, all CHR-P participants in NAPLS 2 were help-seeking individuals referred by health care providers, educators, or social service agencies, or self-referred in response to community education efforts. The most common referral sources were self-referral, referrals via family or friend, and referrals from community providers working with youth in an acute mental health condition that may be partially explained by attenuated psychotic symptoms (Addington et al. 2012). All participants provided consent (or assent with parental consent when appropriate) in accordance with guidelines and regulations at the participating sites of the NAPLS 2 consortium. Demographic characteristics of the present sample are presented in Table 1. Table SI presents characteristics of participants included in the present study and those who were excluded because of unavailable functional and structural MRI data. Figure SI illustrates a flowchart of NAPLS 2 participants by reason for exclusion from the present study.

**Table 1:** Sample characteristics by diagnostic group at baseline

Characteristic	HC (*n* = 142)	CHR-P (*n* = 246)	Statistical test for significance (2-Tailed)	*p* value	Cohen’s *d* (95% CI)
Age, M (SD)	20.18 (4.4)	19.62 (4.0)	*t*(386) = 1.28	0.201	NA
Male sex, No. (%)	78 (54.9)	145 (58.9)	χ^2^(1) = 0.59	0.441	NA
Years of education, M (SD)^a^	12.64 (3.4)	11.64 (2.7)	*t*(240) = 2.98	0.003	0.34 (0.13 to 0.54)
Paternal GED/HS or above, No. (%)	122 (85.9)	205 (83.3)	χ^2^(1) = 0.77	0.679	NA
Maternal GED/HS or above, No. (%)	133 (93.6)	220 (89.4)	χ^2^(1) = 2.05	0.360	NA
Race, No. (%)					
Black/African American	34 (23.9)	44 (17.9)	χ^2^(1) = 2.06	0.152	NA
Central/South American	6 (4.2)	9 (3.7)	χ^2^(1) = 0.08	0.780	NA
East/Southeast/South Asian	15 (10.6)	15 (6.1)	χ^2^(1) = 2.52	0.113	NA
First nations	2 (1.4)	4 (1.6)	χ^2^(1) = 0.03	0.867	NA
Multiracial	10 (7.0)	35 (14.2)	χ^2^(1) = 2.94	0.086	NA
White	74 (52.1)	140 (56.9)	χ^2^(1) = 0.84	0.360	NA
Ethnicity, No. (%)					
Hispanic/Latino	23 (16.2)	47 (19.1)	χ^2^(1) = 0.52	0.473	NA
SIPS negative symptoms, M (SD)^a^	1.53 (2.1)	11.35 (6.4)	*t*(322) = − 22.2	< 0.001	− 1.88 (− 2.13 to − 1.64)
SIPS positive symptoms, M (SD)^a^	1.05 (1.6)	11.80 (3.7)	*t*(363) = − 39.2	< 0.001	− 3.43 (− 3.75 to − .11)

*HC* healthy controls, *CHR-P* clinical high-risk for psychosis, *CI *confidence interval, *M* mean, *SD* standard deviation, *GED* General Educational Development, *HS* high school, *SIPS* Structured Interview for Psychosis-risk Syndromes

^a^Welsh 2-sample *t*-tests were performed when Levene’s test was significant for homogeneity of variance between groups at *p* < 0.05

### Measures

#### Imaging paradigm and data acquisition

Participants underwent a 5-min eyes-open rsfMRI scan with 154 whole brain volumes. At all sites, functional images were collected using gradient-recalled-echo echo-planar imaging sequences: 220 mm isotropic field of view, TR/TE = 2000/30 ms, 77-degree flip angle, 30 4 mm slices, 1 mm gap. Preprocessing was performed using the standard procedures implemented in Statistical Parametric Mapping software, Version 12 (Friston et al. 1995). Specifically, all images were slice-time corrected, realigned for head motion, registered to the individual T1-weighted structural images, and spatially normalized to the Montreal Neurological Institute template with a resampled 2 mm isotropic voxel size. Normalized images were spatially smoothed with an 8 mm full width at half-maximum Gaussian kernel. After preprocessing, BOLD signal time series for each brain region defined by the Automated Anatomical Labeling atlas (Tzourio-Mazoyer et al. 2002) were extracted and corrected for white matter and cerebrospinal fluid signals, 24 head motion parameters (specifically, six rigid-body parameters generated from the realignment step, their first derivatives, and the squares of these 12 parameters), and framewise displacement (Power et al. 2014), and then bandpass filtered at 0.008–0.1 Hz (Cao et al. 2020). Participants with mean framewise displacement > 0.5 mm were excluded from analysis.

At all sites, high-resolution T1-weighted structural images were acquired in the sagittal plane with a 1 mm x 1 mm in-plane resolution and 1.2 mm slice thickness (Cannon et al. 2014). Sequence parameters for acquisition were optimized for scanner manufacturer, software version, and head coil configuration according to the ADNI protocol (summarized in Supplemental materials). Structural images were processed using the Human Connectome Project’s minimal preprocessing pipelines (Glasser et al. 2013). Automatic whole-brain segmentation and surface-based cortical reconstruction were conducted with FreeSurfer version 5.2 (Fischl 2012).

Subcortical brain regions were automatically segmented from structural images using FSL First (Patenaude et al. 2011). Briefly, 2-stage linear registrations were applied to bring the subcortical regions of individual scans into alignment with the Montreal Neurological Institute 152 template space. Next, based on a large training dataset of manually labeled brain images, automatic segmentation was performed to segment subcortical structures. Between-site intraclass correlations showing reliability of subcortical volumes across NAPLS 2 as demonstrated in the NAPLS traveling subjects study (Cannon et al. 2014) are reported in Supplemental materials.

All analyses in the present study were conducted with both raw regional volumes and volumes adjusted for estimated intracranial volume (eICV) using the residual method (Nordenskjöld et al. 2015). On the one hand, for example, HV has been shown to be correlated with eICV (van Eijk et al. 2020). On the other hand, removing variance associated with eICV from subcortical volumes implies that absolute brain size is unimportant. This decision can be questioned conceptually, especially given evidence that larger brain size is protective in neurodegenerative disease states (Guo et al. 2013; Perneczky et al. 2010). More broadly, using raw volumes may be seen as advantageous for reproducibility and cross-study comparisons in a literature confounded by sample differences and varied correction techniques (Lotze et al. 2019).

### Statistical analyses

In the present study, we employ Group Iterative Multiple Model Estimation (GIMME) (Gates and Molenaar 2012) to estimate temporoparietal effective connectivity parameters in people at CHR-P and HCs. GIMME is a data-driven approach that unifies structural equation modeling and vector autoregression to build directed functional network models including autoregressive (same variable across time), contemporaneous (different variables within time), and lagged (different variables across time) parameters (Gates et al. 2010). The procedure has been explicated in a seminal paper describing its appropriateness for application with data from fMRI (Gates and Molenaar 2012) and an accompanying technical paper has been published (Beltz and Gates 2017).

Briefly, the procedure begins with generating a unified structural equation model for each participant in the sample. The individual-level model-building process begins with the estimation of autoregressive parameters for all brain regions selected for analysis, which ensures that remaining contemporaneous and lagged parameters are relevant over and above the self-predicting nature of BOLD signal time series (Shinn et al. 2023). Specifically, to estimate individual-level autoregressive parameters, each instance of BOLD signal is regressed on its own prior value where X_t_ = ΦX_t-1_ + ε_t_ and Φ is a single autoregressive coefficient that accommodates the complete BOLD signal time series. Conceptually, this is similar to the estimated value of slope in a regression model, which accommodates many observations of a single variable.

After autoregressive parameters have been estimated, the residual variance (or how much X_t_ is not explained by X_t-1_) is stabilized for each brain region. Next, remaining contemporaneous (reflecting simultaneous BOLD signal fluctuations) and lagged (e.g., X_t-1_ → Y_t_) interregional parameters are evaluated agnostically. Critically, although contemporaneous parameters are meant to capture simultaneous covariance, unified structural equation modeling cannot estimate true bidirectional covariance when also simultaneously estimating lagged parameters because structural equation modeling requires a non-circular system (Kim et al. 2007). Indeed, to maintain an identifiable structural model that does not violate path tracing rules, GIMME forces the model to choose a direction for contemporaneous parameters according to improvements in model fit (Beltz and Gates 2017; Gates and Molenaar 2012). Residual variance is recalculated after the addition of each subsequent parameter until adequate model fit has been obtained for all individual-level models as evidenced by each individual model meeting the threshold on two out of four canonical fit statistics commonly leveraged in structural equation modeling (i.e., CFI ≥ 0.95, NNFI ≥ 0.95, RMSEA ≤ 0.10, SRMR ≤ 0.05) (Cangur and Ercan 2015; Gates and Molenaar 2012). We emphasize that initial individual model fit does not guarantee individual-level “good fit” when the group-level model is later estimated for all people; specifically, to establish a representative model, not unlike a sample average, all parameters that significantly improved model fit for 75% of individual-level models are estimated for all participants (Gates and Molenaar 2012). This threshold can be adjusted; however, it is commonly used in fMRI studies that attempt to identify a model that fits most individuals in the presence of noise (van Den Heuvel and Sporns 2013) and mitigates the potential for false positives (Beltz and Gates 2017).

To accommodate the likelihood that the 75% group-level threshold misses other estimates relevant to subsamples, GIMME also includes unsupervised (Gates et al. 2017) and supervised clustering procedures (Henry et al. 2019). These procedures are implemented separately but simultaneously with commands that generate individual- and group-level models. Simulation studies of the GIMME procedure have demonstrated excellent parameter recovery within directed functional networks of up to 20 variables (Gates and Molenaar 2012). Regional BOLD signal is sufficiently dissimilar across the cortical hemispheres, especially in temporoparietal regions (Igelström and Graziano 2017; Damoiseaux et al. 2006), so our selection was based on careful consideration of our previous work (Aberizk et al. 2023) and theory concerning the temporoparietal areas that constitute hub regions in brain-wide functional networks (Buckner and DiNicola 2019; Crittenden et al. 2015).

#### Supervised clustering

Complete time series of BOLD signal from ROIs chosen for the GIMME analysis—bilateral hippocampus, inferior parietal lobule, parahippocampal gyrus, posterior cingulate cortex, superior temporal pole, and thalamus—were selected for each participant. These data were provided to the gimme function of the *gimme* package (Lane et al. 2024) in concatenated long form to create a list in R version 4.4 with a vector of diagnostic group identities.

#### Unsupervised clustering

The setup for unsupervised clustering mirrored the supervised approach except that a vector of diagnostic group identities was not provided. Instead, clusters were formed according to the agglomerative Walktrap community detection procedure (Pons and Latapy, 2006; Smith et al. 2020). In principle, a similarity matrix—in which people are “nodes” represented as vectors and elements contain raw counts of shared features, or significant connectivity parameters of the same direction and type (contemporaneous or lagged estimates) between pairs relevant to a given element of the matrix—is split into individual vectors and then merged iteratively to create a dendrogram. In this context, each “merge” is represented by an updated modularity score that directly quantifies the total weight of within-cluster shared connectivity parameters minus the total weight of between-cluster parameters after controlling for counts that may occur by chance. To accommodate chance, sparsity is first introduced in the similarity matrix by setting the lowest integer of raw counts of shared parameters between any two individuals to zero. Walktrap continues to merge participants into larger clusters until maximum modularity has been reached. Walktrap outperforms other approaches in the case of small subgroups (Gates et al. 2017) and has been leveraged in published work using the GIMME procedure with fMRI data (McCormick et al. 2019).

After clusters were generated, GIMME estimated parameters within clusters if they significantly improved model fit for 75% of people in each cluster. The stability of the unsupervised cluster solution was evaluated using the perturbR function of the *perturbR* package (Gates et al. 2019). The objective of this procedure is to randomly alter the counts in the original similarity matrix via random replacement to generate a rewired matrix with the same properties as the original. This process was repeated 100 times at each level of perturbation, or 1–100% of edges perturbed. Unsupervised clusters were compared on proportion of individuals at CHR-P as well as positive and negative symptom totals to determine whether the unsupervised clusters differed in clinical features; in addition, clusters were compared on bilateral HV, amygdala, nucleus accumbens, and thalamus volumes post hoc. All tests were subjected to an adjusted Bonferroni correction at *p* < 0.025 to accommodate repeated comparisons in lateralized regional volumes.

## Results

### Supervised clustering

**Fig. 1:**
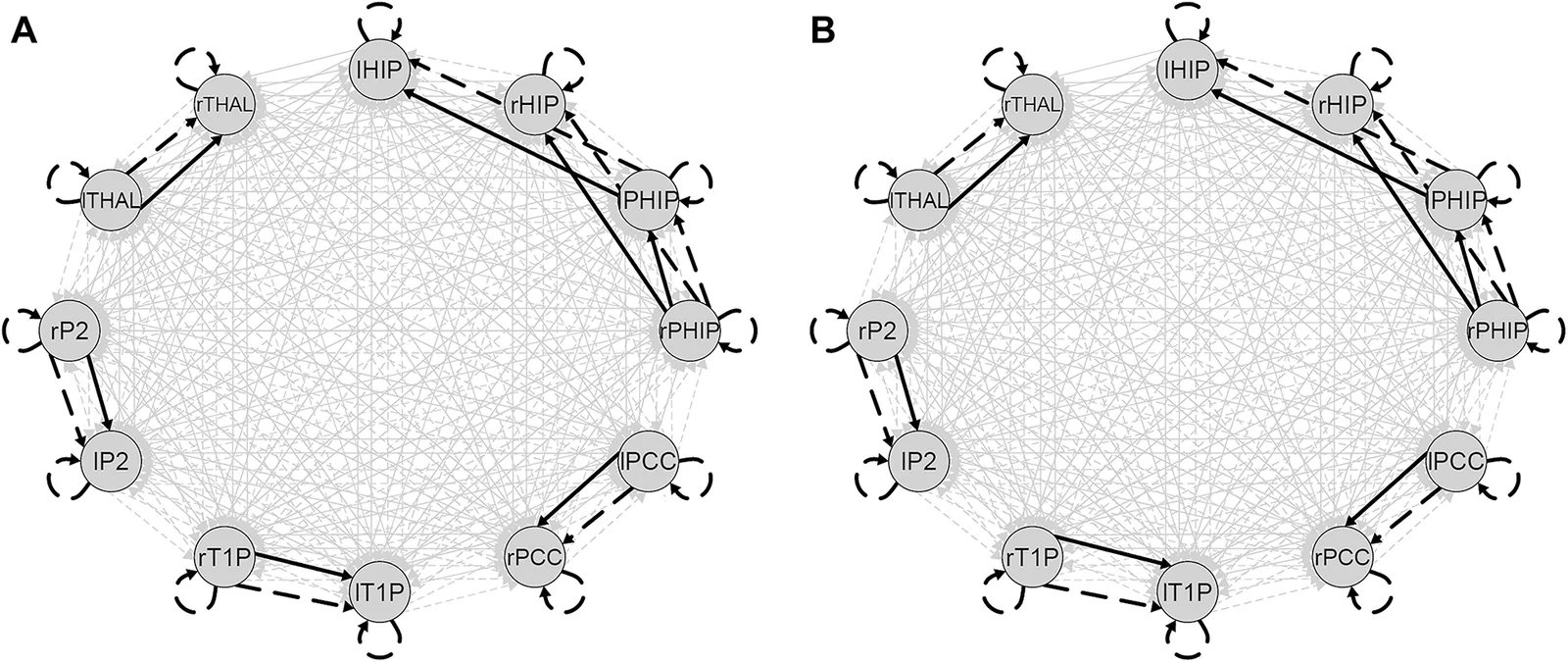
Effective connectivity maps for healthy controls and participants at clinical high-risk for psychosis. *A* = Effective connectivity map for healthy controls; *B* = Effective connectivity map for participants at clinical high-risk for psychosis. Black lines indicate group-level parameters estimated for the full sample at the 75% majority threshold. Grey lines indicate individual-level parameters that did not reach that threshold for group-level estimation. Line thickness reflects proportion of individuals who saw significant improvements in model fit with parameter estimation. Solid lines indicate contemporaneous parameters, and dashed lines indicate lagged parameters. *HIP* hippocampus, *PHIP* parahippocampal gyrus, *PCC* posterior cingulate cortex, *T1P* superior temporal pole, *P2* inferior parietal lobule, *THAL* thalamus

The group-level model (which includes only connectivity parameters relevant to 75% of the sample) is shown in Fig. 1 as the solid black lines shared across the diagnostic groups. When estimated for all individuals, the group-level model achieved modest overall fit according to summary fit statistics provided here as the average (and range) of each fit statistic: RMSEA = 0.17 (0.08–0.22); SRMR = 0.07 (0.02–0.13), NNFI = 0.78 (0.60–0.92), and CFI = 0.86 (0.73–0.96). Across participants, the group-level model achieved excellent model fit for 5.2% of the total sample (as evidenced by meeting threshold on 3 out of 4 canonical fit statistics; Wolf et al. 2016) and adequate model fit for 13.7% of the sample (as evidenced by meeting 2 out of 4 thresholds). Density plots of all fit statistics are shown in Fig. SII.

The following contemporaneous (simultaneous) parameters were included in the group model: left parahippocampal gyrus to left hippocampus, right parahippocampal gyrus to left parahippocampal gyrus and right hippocampus, left posterior cingulate cortex to right posterior cingulate cortex, left thalamus to right thalamus, right inferior parietal lobule to left inferior parietal lobule, and right superior temporal pole to left superior temporal pole. Thus, the most common patterns of temporoparietal effective connectivity among the full sample were cross-hemispheric directed parameters within a brain region. Lagged (across time) parameters of the same direction, and including the same regions, were also generated, as well as autoregressive parameters for all regions. Group-level estimation of these parameters demonstrates their significance to improvements in model fit for at least 75% of all participants.

The supervised clustering procedure generated clusters defined by diagnostic group, i.e., HC or CHR-P. However, supervised clustering did not identify any diagnostic group-specific connectivity parameters, suggesting an absence of any single parameter that would have significantly improved model fit for 75% of either HC or CHR-P participants over and above parameters estimated in the full sample. Here, we note that HC and CHR-P participants did not differ in mean framewise displacement during rsfMRI acquisition in the present study, *t*(386) = -1.42, *p* = 0.158. In addition, a post hoc analysis exclusive to participants at CHR-P—which generated clusters defined by those who did (*n* = 27) and did not (*n* = 219) develop frank psychosis between baseline and the 2-year follow-up in NAPLS 2—did not reveal the relevance of any subgroup-specific connectivity parameters (Fig SIII). Moreover, the group-level connectivity parameters, shown in solid black lines in Fig SIII, are identical to those generated in supervised clustering with the HC and CHR-P subgroups (Fig I), indicating stable features of temporoparietal effective connectivity regardless of CHR-P status.

### Unsupervised clustering

The unsupervised clustering procedure generated two clusters. Results of the perturbation procedure that tested the stability of the unsupervised cluster solution are explained in Supplemental materials. Briefly, identifying which level of edge perturbation (or “rewiring” of a similarity matrix; see Methods and Materials) produces a solution with cluster assignments that are as different as when 10% and 20% of cluster assignments are randomized provides insight into interpretation. Previous work has considered cluster solutions to be robust if > 20% of edges are perturbed before the difference in cluster assignments “intersects” the point when 20% of people in the sample have cluster assignment randomized (Gates et al. 2016; Karrer et al. 2008).

In the present study, the cluster solution is robust as evidenced by the finding that 33% of shared and significant connectivity parameters (counts from the original similarity matrix) needed to be perturbed before the solution was as different as when 20% of participants in the sample had their cluster assignment randomized (Fig SIV). At the same time, we acknowledge that the modularity of our cluster solution is only marginally greater than the modularity values obtained for solutions from random matrices with the same dimensions as our original similarity matrix (Fig SV).

The unsupervised clusters differed significantly in right HV and both positive and negative symptom totals. We observed small-to-medium effect sizes for raw and eICV-adjusted right HV, indicating that Cluster 1 displayed reduced HV relative to Cluster 2. In addition, we observed a small effect size for positive symptoms and small-to-medium effect size for negative symptoms, indicating that Cluster 1 reported greater symptoms relative to Cluster 2. There were no significant differences between unsupervised clusters in age, *t*(386) =-0.81, *p* = 0.418, biological sex, χ^2^(1) = 0.84, *p* = 0.360, or CHR-P status, χ^2^(1) = 0.71, *p* = 0.398. Post hoc, we conducted *z-*tests to compare effect sizes for differences in positive and negative symptoms between unsupervised clusters and between HC and CHR-P. Relative to unsupervised cluster assignment, CHR-P status more strongly distinguished participants based on positive symptoms, *z* = 2.55, *p* = 0.010, yet there was no significant difference in effect size for differences in negative symptoms, *z* = 1.55, *p* = 0.122. Mean difference tests of clinical and neuroanatomical features by cluster membership are shown in Table 2.

**Table 2:** Clinical and neuroanatomical characteristics by unsupervised cluster membership

Characteristic	Cluster 1 (*n* = 275)	Cluster 2 (*n* = 113)	Statistical test for significance (2-Tailed)	*p* value	Cohen’s *d* (95% CI)
SIPS negative symptoms, M (SD)^a^	8.4 (7.2)	6.0 (6.2)	*t*(241) = 3.28	0.001*	0.35 (0.12 to 0.57)
SIPS positive symptoms, M (SD)	8.3 (6.1)	6.8 (5.7)	*t*(386) = 2.32	0.021*	0.26 (0.04 to 0.48)
Raw volumes					
Left accumbens (mm^3^), M (SD)	738 (138)	733 (156)	*t*(386) = 0.32	0.751	NA
Right accumbens (mm^3^), M (SD)	719 (117)	715 (110)	*t*(386) = 0.34	0.736	NA
Left amygdala (mm^3^), M (SD)	1690 (218)	1726 (231)	*t*(386) = − 1.45	0.147	NA
Right amygdala (mm^3^), M (SD)	1771 (260)	1811 (252)	*t*(386) = − 1.39	0.166	NA
Left hippocampus (mm^3^), M (SD)	4171 (491)	4247 (452)	*t*(386) = − 1.42	0.158	NA
Right hippocampus (mm^3^), M (SD)	4198 (483)	4338 (453)	*t*(386) = − 2.65	0.009*	− 0.30 (− 0.52 to − 0.08)
Left thalamus (mm^3^), M (SD)	7919 (948)	8130 (969)	*t*(386) = − 1.98	0.048	NA
Right thalamus (mm^3^), M (SD)	7399 (870)	7527 (881)	*t*(386) = − 1.32	0.189	NA
eICV-adjusted volumes					
Left accumbens (mm^3^), M (SD)	739 (133)	731 (154)	*t*(386) = 0.54	0.591	NA
Right accumbens (mm^3^), M (SD)	720 (108)	712 (110)	*t*(386) = 0.65	0.515	NA
Left amygdala (mm^3^), M (SD)	1693 (186)	1718 (205)	*t*(386) = − 1.18	0.238	NA
Right amygdala (mm^3^), M (SD)	1775 (205)	1801 (220)	*t*(386) = − 1.09	0.275	NA
Left hippocampus (mm^3^), M (SD)	4178 (401)	4229 (387)	*t*(386) = − 1.13	0.256	NA
Right hippocampus (mm^3^), M (SD)	4206 (358)	4317 (373)	*t*(386) = − 2.74	0.006*	− 0.31 (− 0.53 to − 0.09)
Left thalamus (mm^3^), M (SD)	7938 (675)	8085 (687)	*t*(386) = − 1.94	0.053	NA
Right thalamus (mm^3^), M (SD)	7415 (629)	7487 (690)	*t*(386) = − 1.00	0.316	NA

*SIPS* Structured Interview for Psychosis-risk Syndromes, *M* mean, *SD* standard deviation, *eICV* estimated intracranial volume, *CI* confidence interval

^a^Welsh 2-sample *t*-tests were performed when Levene’s test was significant for homogeneity of variance between clusters at *p* < 0.05

*Significant after correction for multiple comparisons at *p* < 0.025

We also observed a small-to-medium effect size for a difference between unsupervised clusters in mean framewise displacement during rsfMRI acquisition, *t*(386) = -3.04, *p* = 0.003, *d* = 0.34, indicating that Cluster 1 moved less than Cluster 2 during rsfMRI. Following results of the mean difference tests, multiple regression procedures regressed unadjusted and eICV-adjusted right HV against age, biological sex, mean framewise displacement, and cluster membership. Findings presented in Table 3 demonstrate a significant main effect of cluster in the prediction of right HV. Finally, Table SII demonstrates the means and standard deviations of the variability in regional BOLD signal as evidenced by standard deviations of the time series between unsupervised clusters. Cluster 1 displayed significantly less variability in BOLD signal relative to Cluster 2.

**Table 3:** Results of multiple regressions of right hippocampal volume against age, biological sex, mean framewise displacement, and unsupervised cluster membership

	beta	SD	*t* value	*p* value	95% CI	
					LL	UL
Right HV						
Age	− 5.37	5.27	− 1.02	0.309	− 15.72	4.99
Sex (M)	419.25	43.89	9.55	< 0.001	332.96	505.55
Mean FD	− 366.53	258.99	− 1.42	0.158	− 875.76	142.70
Cluster (2)	131.66	48.34	2.72	0.007*	36.62	226.71
eICV-adjusted right HV						
Age	1.18	4.47	0.27	0.791	− 7.60	9.97
Sex (M)	60.97	37.22	1.64	0.102	− 12.21	134.15
Mean FD	− 322.64	219.63	− 1.47	0.143	− 754.47	109.20
Cluster (2)	116.66	40.99	2.85	0.005*	36.07	197.26

*HV* hippocampal volume, *M* male, *FD* framewise displacement, *SD* standard deviation, *CI* confidence interval, *LL* lower level, *UL* upper level. Reference level for categorical variables indicated parenthetically

*Significant after Bonferroni correction at *p* < 0.025 to accommodate repeated comparisons in lateralized hippocampal volume

**Fig. 2:**
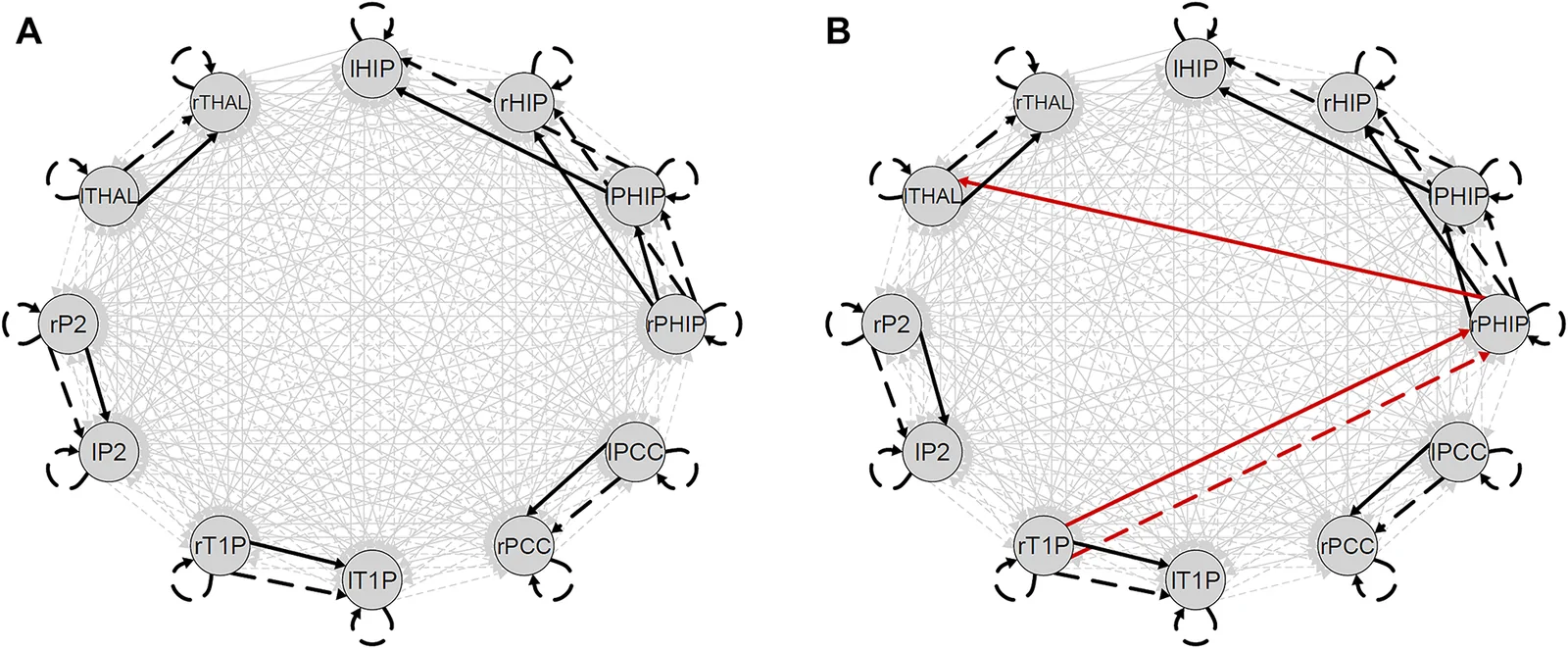
Effective connectivity maps for unsupervised clusters. *A* = Effective connectivity map for cluster with greater symptoms and smaller hippocampal volume; *B* = Effective connectivity map for cluster with fewer symptoms and larger hippocampal volume. Black lines indicate group-level parameters estimated for the full sample at the 75% majority threshold. Red lines indicate cluster-level parameters estimated for the second cluster at the 75% majority threshold. Grey lines indicate individual-level parameters that did not reach those thresholds for estimation. Line thickness reflects proportion of individuals who saw significant improvements in model fit with parameter estimation. Solid lines indicate contemporaneous parameters, and dashed lines indicate lagged parameters. *HIP *hippocampus, *PHIP *parahippocampal gyrus, *PCC *posterior cingulate cortex, *T1P* superior temporal pole, *P2* inferior parietal lobule, *THAL *thalamus

The cluster including participants with reduced right HV was also characterized by greater positive and negative symptoms (*n* = 275). However, as shown in Fig. 2, effective connectivity for that cluster was not characterized by any cluster-specific parameters, suggesting the lack of any single parameter that would have significantly improved model fit for a 75% majority of that cluster. In contrast, effective connectivity for the cluster with greater right HV and fewer symptoms (*n* = 113) showed contemporaneous estimates from the right superior temporal pole to right parahippocampal gyrus and right parahippocampal gyrus to left thalamus; in addition, this cluster promoted a lagged estimate from the right superior temporal pole to the right parahippocampal gyrus (Fig. 2).

## Discussion

The present study demonstrated the relevance of variability in right HV to temporoparietal effective connectivity in a sample of HCs and youth at CHR-P. Indeed, right HV emerged as a significant factor that discerned latent clusters established according to common patterns of temporoparietal effective connectivity. These findings are viewed as consistent with our previous work demonstrating associations of HV and static hippocampal *FC* (Aberizk et al. 2023) and other reports of significant relations of HV with DMN-temporoparietal FC (De Marco et al. 2019; Seidman et al. 2014). In the present study, participants did not self-organize by CHR-P status during unsupervised clustering. Instead, the same cluster demonstrating reduced right HV reported greater attenuated positive and negative symptoms.

The supervised clustering procedure that organized participants by CHR-P status did not uncover any single connectivity parameter relevant to a 75% majority of either HC or CHR-P groups over and above the parameters estimated in the full sample. Further, the supervised procedure exclusive to participants at CHR-P, which required cluster organization by prospective conversion to psychosis status, did not uncover any single effective connectivity parameter relevant to the majority of either converters or nonconverters. However, it is important to emphasize the observed consistency of the group-level connectivity parameters in the common functional network generated across both supervised procedures. Group estimates were identical across iterations and indicate stable features of temporoparietal effective connectivity regardless of CHR-P status. Indeed, CHR-P status may only relate to group differences in these brain regions under certain circumstances (e.g., stress induction or emotional memory tasks; Pauly et al. 2010). More broadly, most individuals who meet criteria for CHR-P will not develop psychosis (Salazar de Pablo et al. 2022), so heterogeneity in the CHR-P sample may compromise the detection of a homogenous subgroup distinguishable from HCs.

During unsupervised clustering, when participants self-organized based on commonalities in their individual patterns of data, clusters were discernable by right HV. There is evidence from the literature on neurodegenerative diseases to suggest that right HV is relevant to patterns of brain connectivity. For example, in Alzheimer’s disease, which is characterized by significant reductions in HV, evidence suggests stronger associations of brain functional network topology with right HV than left HV (Vecchio et al. 2017). Right HV also demonstrates greater plasticity than left HV in humans and non-human animals (Esteves et al. 2021). Further, adversity during childhood has been associated with reduced right HV, and not left HV, in large samples of healthy youth (Breslin et al. 2024; Ku et al. 2024). Critically, it is important to emphasize that reduced HV observed in one cluster of participants in the present study is not necessarily pathological. However, the same cluster demonstrating significantly smaller right HV also reported greater attenuated positive and negative symptoms in the present study.

Negative symptoms are neither necessary nor sufficient for a diagnosis of psychotic disorder or designation of CHR-P status, and the comorbidity of negative symptoms (e.g., with major depressive or trauma-related disorders) has inspired decades of research (Gupta et al. 2021; Kirkpatrick et al. 2023). Indeed, negative symptoms significantly predict functional and prognostic outcomes with improvements in anhedonia, avolition, and asociality being rated as important to recovery among affected individuals (Strauss et al. 2026). Critically, relative to unsupervised cluster membership, CHR-P status more strongly distinguished participants in the present study based on reports of positive symptoms. However, there was no significant difference in the effect size for a difference in negative symptoms based on CHR-P status or unsupervised cluster membership. It is not uncommon to find differences in personality or global functioning when individuals are allowed to self-organize by common patterns of resting-state connectivity. For example, a recent study found unsupervised clustering to reveal five clusters (one exclusive to HCs) that differed significantly in personality features (Zwir et al. 2023). In light of evidence that temporoparietal brain regions are implicated in self-referential thinking, including evaluations of the self in a larger context (Andrews-Hanna et al. 2010; Buckner and DiNicola 2019; Crittenden et al. 2015), it is perhaps unsurprising that clustering by temporoparietal effective connectivity produced subsamples that differ in negative symptom severity, which involves evaluations of the self with regard to sociability, motivation, and affective expression.

Specifically, effective connectivity within the cluster with greater right HV and fewer symptoms was characterized by significant modulation from the superior temporal pole as evidenced by the directed paths, both contemporaneous and lagged, from the superior temporal pole to hippocampal formation. The hippocampal formation and temporal pole are known to coordinate the retrieval of different memory representations for coherent autobiographical recall (Setton et al. 2022). Moreover, modulation from the temporal pole increases as cues converge to indicate a probable psychosocial outcome (Pehrs et al. 2017). In contrast, decreased network centrality in the hippocampal formation and temporal pole is associated with cognitive impairment (Qiu et al. 2016). Additional research is warranted to examine how activation in the temporal pole interacts with autobiographical and episodic memory performance, which may be relevant to the expression of attenuated positive and negative symptoms.

Longitudinal studies are also warranted to address the issue of temporal precedence in structure–function coupling and indicate the long-term coherence of observed relations. In addition, alongside the burgeoning literature on structure–function coupling (Fotiadis et al. 2024), novel methods for the comprehensive examination of structure–function coupling have been developed in recent years. For example, some investigators have developed effective connectivity techniques that accommodate a brain-wide network perspective (Mellema and Montillo 2023; Cole et al. 2021) while others have suggested that, in the context of structure–function coupling, the gray-to-white matter boundary be considered as a structural feature consistent with evidence that neural activity originates in gray matter (Weinstein et al. 2024). We view our focus on subcortical gray matter volumes as consistent with the latter point. More broadly, although several challenges are associated with translating findings from conventional high-field MRI to clinical and community settings (e.g., installation and maintenance costs, space requirements), recent work using portable low-field MRI systems has demonstrated moderate correspondence for subcortical gray matter volume measures obtained from high- vs low-field MRI (Cooper et al., 2024). Study designs that leverage these low-field technologies are warranted to determine whether inferences drawn from studies using high-field systems can be translated into clinical practice for early detection or prevention.

The present study has limitations. First, participants at CHR-P drive the substantive inferences of the present study as both unsupervised clusters contain a majority of participants at CHR-P. However, CHR-P participants excluded from the present study for unavailable functional and structural MRI data (Table SI) differed from included participants at CHR-P (e.g., lower years of education in excluded participants), which limits the generalizability of our findings. Further, it is tempting to speculate that Cluster 1 (smaller right HV, greater symptoms) is a “catch all” cluster, which may partially explain the lack of cluster-specific connectivity parameters in Cluster 1, yet Cluster 1 demonstrated significantly reduced variability in regional BOLD signal (as evidenced in smaller standard deviations of their time series; Table SII) and lower framewise displacement during rsfMRI acquisition relative to Cluster 2.

Further, our findings do not shed light on brain functional network dynamics beyond the temporoparietal regions selected for analysis. Our selection was based on careful consideration of our previous work (Aberizk et al. 2023), established limitations of the GIMME procedure (Gates and Molenaar 2012), and theory concerning the temporoparietal regions that constitute hub regions in brain-wide functional networks (Buckner and DiNicola 2019). Still, it is important to acknowledge that our selection obscures more nuanced features of network dynamics and their relation to brain structural characteristics or global functioning. For example, hippocampal effective connectivity with the ventromedial prefrontal cortex has been implicated in both stress susceptibility and resilience. A recent study demonstrated that people with stronger connectivity between those regions endorsed less stressful feelings at the outset of the COVID-19 pandemic *and* lower levels of resilience five months later. Specifically, effective connectivity among those with delayed susceptibility to stress lacked a directed path from the hippocampus to the ventromedial prefrontal cortex, which was hypothesized to compromise resiliency (Chang et al. 2023).

 In addition, little is known about the relation of psychotropic medication use with patterns of brain connectivity (Duan et al. 2020). Many people at CHR-P have had previous treatment with one or more of these classes of medication (Woods et al. 2013). For ethical reasons, people who meet CHR-P criteria and participate in research are not required to stop current medications. Thus, statistically controlling for medication type or dosage would be challenging, especially in light of the various psychotropic subtypes, dosages, and changes to adherence that may or may not be documented. Finally, the short length of the resting-state time series is also a notable limitation of the present study. Indeed, longer time series may be better powered to detect group differences (e.g., HC or CHR-P groups) or yield additional or alternate individual-level effective connectivity parameters that ultimately affect the group-level model in GIMME. Although many reported applications of GIMME used time series of 100 or fewer measurements (Beltz and Gates 2017), test–retest reliability of rsfMRI connectivity estimates have been shown to improve significantly by increasing scan length to ≥ 10 min with improvements plateauing around 15 min (Birn et al. 2013).

In conclusion, we provide novel evidence suggesting that right HV may be relevant to temporoparietal effective connectivity. These findings advance our recent work with this sample, which demonstrated the relevance of seed-based hippocampal FC with these temporoparietal regions in relation to HV (Aberizk et al. 2023). By leveraging the flexible clustering procedures available in GIMME (Gates et al. 2017; McCormick et al. 2019; Henry et al. 2019), we offer a foundation for further theorizing about the coherence between brain functional network topology and the integrity of structural features that anchor those networks. The growing consensus and evidence for neurobiological heterogeneity within and across categories of psychotic disorders and other psychiatric illnesses suggests that heterogeneity is a practical assumption in clinical research (Keane et al. 2024; Walker and Goldsmith 2022). Indeed, heterogeneity may be an opportunity to identify biologically meaningful subgroups. Critically, no single mechanism can account for brain functional or structural alterations in any psychiatric disorder (Hall et al. 2015). Future research that aims to advance this important area of investigation must clarify the presently insufficient longitudinal evidence (Holz et al. 2023) for normative structure–function coupling mechanisms.

## Supplementary Information

Below is the link to the electronic supplementary material.

Supplementary file1 (DOCX 810 KB)

## Data Availability

The datasets generated and analyzed during the current study are not publicly available but are available from the corresponding author on reasonable request.
