# Supplementary material for: Right hippocampal volume and attenuated psychotic symptoms distinguish subgroups of youth with common patterns of temporoparietal effective connectivity
Source: Brain Struct Funct. 2026 Aug 8;231(7):117. doi: 10.1007/s00429-026-03161-1 (PMC13452762; doi:10.1007/s00429-026-03161-1)
Supplement: Supplementary file 1 [file 429_2026_3161_MOESM1_ESM.docx]

**Supplemental Materials**

**Imaging Acquisition Parameters in NAPLS 2**
All eight sites in NAPLS 2 operated 3 Tesla scanners. Five sites operated Siemens scanners and three sites operated GE scanners. All Siemens sites used a 12-channel head coil and all GE sites used an 8-channel head coil. Sequence parameters were optimized for scanner manufacturer, software version, and coil configuration according to the ADNI protocol. At all sites, scans were acquired in the sagittal plane with a 1mm x 1mm in-plane resolution and 1.2mm slice thickness. Siemens scanners used a magnetization prepared rapid acquisition gradient-echo (MPRAGE) sequence with a 256mm x 240mm x 176mm field of view, TR/TE/TI = 2300/2.91/900ms and a 9-degree flip angle. GE scanners used an inversion recovery spoiled gradient recalled echo (IR-SPGR) sequence (efgre3d_cs) with a 26cm field of view, TR/TE/TI = 7.0/minimum full/400ms and an 8-degree flip angle. The ADNI structural phantom was scanned at each site. All T1-weighted images were inspected visually by at least two highly trained raters to ensure that only high-quality images with no noticeable problems with segmentation and/or surface projection were included in analyses. **Traveling Subjects Study in NAPLS 2**	NAPLS 2 conducted a traveling subjects study to demonstrate within- and between-scanner reliability of neuroanatomical measures. All scanners and acquisition parameters used in the traveling subjects study are as described above. Each of the eight participating sites recruited one healthy participant (4 males, 4 females) between the ages of 20 and 31 (mean = 26.9, SD = 4.3) who was scanned twice on successive days at every site for a total of 128 scans (8 participants x 2 scans x 8 sites). Scanning was conducted between May and August of 2011. There were no equipment or software changes at any site during that period. All participants completed all scans within two months, and the order of visits to sites was counterbalanced.
**Reliability of Volume Measures**
ICCs are a common reliability measure. ICC is calculated as variance of interest divided by total variance, and each variance component is quantified by mean squares, i.e., sum of squares divided by degrees of freedom. Participant, site, and session were fully crossed in the traveling subjects study for NAPLS 2; therefore, the total variance in each regional brain volume was divided into variance attributable to participant, site, session, participant x site, participant x session, site x session, and participant x site x session.
Two types of ICCs were computed: *(1)* fraction of variance attributable to participant, reflecting reliability across sites and sessions, i.e., between-site ICC, and *(2)* fraction of variance attributable to participant and scanner, reflecting reliability across scanning sessions for the same participant at the same site, i.e., within-site ICC. Given that between-site ICC indicates reliability after considering nuisance variance due to scanner, site, and session, we report the between-site ICC. Hippocampal and amygdala volumes demonstrated good reliability across NAPLS 2 sites as evidenced by between-site ICCs of 0.791 and 0.764, respectively, and nucleus accumbens and thalamus volumes demonstrated excellent reliability as evidenced by between-site ICCs of 0.876 and 0.957, respectively.

**Table SI.** Demographic characteristics by unavailability of functional and structural magnetic resonance imaging

| **Characteristic** | **Excluded**  **(*n* = 655)** | **Included**  **(*n* = 388)** | **Statistical Test for Significance (2-Tailed)** | ***p* value** | **Cohen’s *d* (95% CI)** |
| --- | --- | --- | --- | --- | --- |
| **Age, mean (SD)** | 18.61 (4.5) | 19.22 (4.2) | *t*(1041) = - 2.17 | .031 | - 0.14 (- 0.26 – - 0.01) |
| **Male sex, No. (%)** | 354 (54.0) | 223 (57.5) | χ^2^(1) = 1.6 | .282 | NA |
| **CHR-P, No. (%)** | 518 (79.1) | 246 (63.4) | χ^2^(1) = 30.58 | < .001 | NA |
| **Years of Education, mean (SD)** | 11.46 (3.1) | 12.00 (3.0) | *t*(1041) = - 2.75 | .006 | - 0.18 (- 0.30 – - 0.05) |
| **Paternal GED/HS or above, No. (%)** | 539 (82.3) | 327 (84.3) | χ^2^(1) = 1.28 | .528 | NA |
| **Maternal GED/HS or above, No. (%)** | 565 (86.3) | 353 (91.0) | χ^2^(1) = 5.46 | .065 | NA |
| **Race, No. (%)** |  |  |  |  |  |
| **Black/African American** | 88 (13.4) | 78 (20.1) | χ^2^(1) = 8.10 | .004 | NA |
| **Central/South American** | 32 (4.9) | 15 (3.9) | χ^2^(1) = 0.59 | .443 | NA |
| **East/Southeast/South Asian** | 54 (8.2) | 30 (7.7) | χ^2^(1) = 0.09 | .769 | NA |
| **First Nations** | 11 (1.7) | 6 (1.5) | χ^2^(1) = 0.03 | .870 | NA |
| **Multiracial** | 95 (14.5) | 45 (11.6) | χ^2^(1) = 1.33 | .248 | NA |
| **White** | 375 (57.3) | 214 (55.2) | χ^2^(1) = 0.44 | .509 | NA |
| **Ethnicity, No. (%)** |  |  |  |  |  |
| **Hispanic/Latino** | 122 (18.6) | 70 (18.0) | χ^2^(1) = 0.06 | .805 | NA |

*CHR-P* = clinical high-risk for psychosis; *SD* = standard deviation; *GED* = General Educational Development; *HS* = high school.

**Table SII.** Standard deviations of regional BOLD signal time series by unsupervised cluster membership

| **Region** | **Cluster 1**  **(*n* = 275)** | **Cluster 2**  **(*n* = 113)** | **Statistical Test for Significance (2-Tailed)** | ***p* value** | **Cohen’s *d* (95% CI)** |
| --- | --- | --- | --- | --- | --- |
| **L HIP, M (SD)^a^** | 1.77 (1.6) | 2.41 (2.0) | *t*(170) = - 3.02 | .003 | - 0.37 (- 0.59 – - 0.15) |
| **R HIP, M (SD)^a^** | 1.68 (1.5) | 2.30 (1.8) | *t*(173) = - 3.17 | .002 | - 0.39 (- 0.61 – - 0.17) |
| **L PHIP, M (SD)^a^** | 2.51 (2.3) | 3.52 (3.0) | *t*(170) = - 3.23 | .001 | - 0.40 (- 0.62 – - 0.18) |
| **R PHIP, M (SD)^a^** | 2.40 (2.1) | 3.39 (2.8) | *t*(168) = - 3.38 | .001 | - 0.42 (- 0.64 – - 0.20) |
| **L PCC, M (SD)^a^** | 4.38 (3.6) | 5.48 (4.4) | *t*(178) = - 2.36 | .019 | - 0.29 (- 0.51 – - 0.07) |
| **R PCC, M (SD)^a^** | 4.27 (3.5) | 5.36 (4.2) | *t*(177) = - 2.42 | .017 | - 0.29 (- 0.51 – - 0.07) |
| **L T1P, M (SD)^a^** | 3.58 (3.2) | 5.26 (4.7) | *t*(156) = - 3.52 | .001 | - 0.46 (- 0.68 – - 0.24) |
| **R T1P, M (SD)^a^** | 3.19 (2.9) | 4.81 (4.2) | *t*(157) = - 3.78 | < .001 | - 0.49 (- 0.71 – - 0.27) |
| **L P2, M (SD)^a^** | 3.33 (2.8) | 4.62 (3.6) | *t*(168) = - 3.37 | .001 | - 0.42 (- 0.64 – - 0.20) |
| **R P2, M (SD)** | 3.90 (3.3) | 5.08 (3.8) | *t*(386) = - 3.07 | .002 | - 0.34 (- 0.56 – - 0.12) |
| **L THAL, M (SD)^a^** | 2.04 (1.7) | 2.97 (2.3) | *t*(164) = - 3.90 | < .001 | - 0.49 (- 0.72 – - 0.27) |
| **R THAL, M (SD)^a^** | 2.11 (1.7) | 3.09 (2.4) | *t*(160) = - 4.00 | < .001 | - 0.51 (- 0.73 – - 0.29) |

*BOLD* = blood oxygenation level-dependent; *CI*= confidence interval; *HIP* = hippocampus; *PHIP* = parahippocampal gyrus; *PCC* = posterior cingulate cortex; *T1P* = superior temporal pole; *P2* = inferior parietal lobule; *THAL* = thalamus; *M* = mean; *SD* = standard deviation.
^a^Welsh 2-sample *t*-tests were performed when Levene’s test was significant for homogeneity of variance between clusters at *p* < .05.

**Table SIII**. Neuroanatomical characteristics adjusted for eICV and site by unsupervised cluster membership

| **Characteristic** | **Cluster 1**  **(*n* = 275)** | **Cluster 2**  **(*n* = 113)** | **Statistical Test for Significance (2-Tailed)** | ***p* value** | **Cohen’s *d* (95% CI)** |
| --- | --- | --- | --- | --- | --- |
| **Left Accumbens (mm^3^), M (SD)** | 739 (133) | 730 (154) | *t*(386) = 0.57 | .567 | NA |
| **Right Accumbens (mm^3^), M (SD)** | 721 (108) | 712 (110) | *t*(386) = 0.68 | .499 | NA |
| **Left Amygdala (mm^3^), M (SD)** | 1693 (186) | 1718 (205) | *t*(386) = - 1.15 | .250 | NA |
| **Right Amygdala (mm^3^), M (SD)** | 1775 (205) | 1801 (220) | *t*(386) = - 1.10 | .271 | NA |
| **Left Hippocampus (mm^3^), M (SD)** | 4178 (401) | 4229 (387) | *t*(386) = - 1.13 | .259 | NA |
| **Right Hippocampus (mm^3^), M (SD)** | 4206 (358) | 4317 (372) | *t*(386) = - 2.76 | .006* | - 0.31 (- 0.53 – - 0.09) |
| **Left Thalamus (mm^3^), M (SD)** | 7938 (674) | 8086 (689) | *t*(386) = - 1.96 | .051 | NA |
| **Right Thalamus (mm^3^), M (SD)** | 7415 (629) | 7486 (690) | *t*(386) = - 0.98 | .328 | NA |

*eICV* = estimated intracranial volume*; CI* = confidence interval; *M* = mean; *SD* = standard deviation.
*Significant after correction for multiple comparisons at *p* < .025.

**Figure SI.** Flowchart illustrating exclusion by unavailable neuroimaging data

*N* = 1043

Excluded those without structural neuroimaging data
(*n* = 323)

*n* = 720

Excluded those without functional neuroimaging data
(*n* = 319)

*n* = 401

Excluded those with excessive head motion during functional neuroimaging (*n* = 12)

*n* = 389

Excluded those with incomplete resting-state magnetic resonance imaging (*n* = 1)

*n* = 388

**Figure SII.** Distribution of individual-level model fit statistics after group-level model-fitting

*A* = Root Mean Squared Error of Approximation; *B* = Standardized Root Mean Square Residual; *C* = Normed Fit Index; *D* = Comparative Fit Index.

**Figure SIII**. Effective connectivity maps for participants at clinical high-risk for psychosis by conversion status

**B**

**A**

*A* = Effective connectivity map for nonconverters; *B* = Effective connectivity map for converters. Black lines indicate group-level parameters estimated for the full sample at the 75% majority threshold. Solid lines indicate contemporaneous parameters, and dashed lines indicate lagged parameters. *HIP* = hippocampus; *PHIP* = parahippocampal gyrus; *PCC* = posterior cingulate cortex; *T1P* = superior temporal pole; *P2* = inferior parietal lobule; *THAL* = thalamus.

**Figure SIV**. Comparison of original result against perturbed similarity matrix

*VI* = variation of information. Proportion perturbed reflects how many edges were disrupted in the original similarity matrix, and mean variation of information reflects the degree of difference in cluster assignments between the original similarity matrix and the rewired matrix. Black dots represent the average difference value between the original and rewired matrices across iterations (*n* = 100 bootstrapped samples) at each level of disturbance with error bars indicating two standard errors above and below the mean. Red dots represent the same differences values on a random matrix with the same number of elements as a null for comparison. Horizontal lines reflect the difference value when 10% and 20% of people in the sample had their cluster assignment randomized. In the figure, black dots cross the 20% line at 33% of perturbation.

**Figure SV**. Distribution of modularity values obtained for solutions in random matrices simulated to have properties similar to the original similarity matrix

Red line indicates the modularity score obtained for the cluster solution in the present study.
